# Supplementary material for: Phosphorylation: new star of pathogenesis and treatment in steatotic liver disease
Source: Lipids Health Dis. 2024 Feb 17;23:50. doi: 10.1186/s12944-024-02037-9 (PMC10873984; doi:10.1186/s12944-024-02037-9)
Supplement: Supplementary file 1 — Additional file 1. 17718536_TiansuLv.docx [file 12944_2024_2037_MOESM1_ESM.pdf]

17718536_TiansuLv.docx

WORD COUNT 10452

TIME SUBMITTED 15-JAN-2024 02:35PM

PAPER ID 106154421

Phosphorylation: New star of pathogenesis and treatment in steatotic liver disease

Tiansu Lv[1,2], Yan Lou[1], Qianhua Yan[1,2], Lijuan Nie[2], Zhe Cheng[2], Xiqiao Zhou [1,2*]

Introduction

Due to advancements in medicine and changing times, the term nonalcoholic fatty liver disease (NAFLD) is no longer regarded suitable due to its exclusivity and stigma. It has been replaced by metabolic dysfunction-associated steatotic liver disease (MASLD). At 2023 Fidel Consensus Statement of multiple academic groups, it has been suggested to change NAFLD to MASLD [1]. Despite this, the vast majority of patients with NAFLD show consistent progression of MASLD [2, 3]. MASLD and NAFLD remain similar in multiple international cohorts on prevalence and hazard factors [4, 5]. NAFLD, also known as MASLD, is associated with steatotic liver disease (SLD) and is currently estimated to affect roughly one fourth population over the world [6]. SLD involves a spectrum ranging from simple steatosis to steatohepatitis, eventually leading to necroinflammation and accelerated progression of fibrosis, culminating in severe cirrhosis and potentially hepatocellular carcinoma (HCC) [7]. Despite advancements in several clinical trials for SLD, the lack of a comprehensive understanding of its complex pathogenesis and underlying molecular mechanisms has hindered the development of effective therapeutics[8, 9]. Currently, maintaining a healthy lifestyle and achieving weight loss are crucial for prevention and treatment, but in reality, they are not sufficient.

Essentially, proteins are the primary agents of life activities and play a role in regulating diseases. In SLD, various proteins can participate in lipid regulation to influence the development of SLD, such as transmembrane 6 superfamily member 2 (TM6SF2), which is relatively necessary for lipidosis of very-low-density-lipoprotein in the Pre-Golgi[10], and widely involved in NAFLD and other cardiovascular diseases [11]. However, simply researching protein levels seems insufficient for today's needs. By further exploring the protein structure at amino acid sites using high-throughput technology, it may be possible to investigate the deeper and more direct mechanisms of SLD. Phosphorylation as one common modification has been studied wide. It is the process of adding phosphate groups to intermediate metabolites or proteins, serving as a major protein modification mechanism [12].

Although phosphorylation may occur on any molecule, it most commonly occurs in regular cases. Over the past few decades, accumulating evidence has validated an essential relationship between phosphorylation and SLD. Notably, due to the complexity of phosphorylation research in SLD, there is currently no comprehensive systematic review available to provide a further summary. However, phosphorylation is closely associated with the development of SLD. Furthermore, numerous medications can enhance SLD by targeting phosphorylation sites. Therefore, the research on phosphorylation in SLD have been summarized, aiming to provide initial insights for SLD, whether NAFLD or MASLD from the perspective of phosphorylated protein modification.

Roles of phosphorylation for SLD

Phosphorylation is an essential cellular process that involves transferring phosphate groups. It

is traditionally regarded as an "on/off switch" that regulates the function of molecules or signaling pathways. In eukaryotes, phosphorylation typically occurs on serine, threonine, and tyrosine residues. In SLD, phosphorylation modifications can be abnormally activated or inhibited by certain triggers, such as nutrition imbalance [13], aging [14], smoking [15], unhealthy diets [16], absent exercise [17], and corresponding metabolic diseases like diabetes [18] or hypertension [19] , disrupting normal physiological activities and promoting SLD. Although it performs similarly in most diseases, it typically involves three aspects: the protein site modified by phosphorylation, the protein kinase that leads to phosphorylation modification, and the phosphatase that performs dephosphorylation. Subsequently, the normal balance of phosphorylation is disrupted, thereby affecting regular life activities. These effects are preliminarily summarized in the following five aspects.

1.Regulation

Phosphorylation or dephosphorylation of a site can activate or inhibit the function of downstream molecules or signaling pathways. This can be referred to as the function of regulation. In general, phosphorylation modification can promote signaling pathways, leading to SLD. However, this is not absolute. Based on the literature summarized, it appears that certain molecules have a dual effect, either activating or inhibiting the downstream pathways in SLD. However, their harmful or protective effects have been identified based on experimental evidence. **Table1** summarized the regulations of the most common molecules on signaling pathways/targets via phosphorylation in SLD. The AMPK, transforming growth factor kinase 1 (TAK1), c-Jun N-terminal kinase (JNK), and others indicate both promotional and inhibitory roles in the downstream targets, which are of great value in SLD.

2.Involving molecule stability

The addition of phosphate groups has been suggested to improve the stability of molecules or metabolites. For example, sphingomyelin phosphodiesterase 3 (SMPD3) is modified by a ubiquitin group, which typically leads to its degradation [20]. When phosphorylated by upstream AMPK, the phosphate group on SMDP3 inhibits ubiquitination, ultimately preventing its degradation and enhancing stability. Another example is the enhancement of protein stability in Inositol 1,4,5-trisphosphate receptor type 1 (IP3R1) through palmitic acid-induced phosphorylation at Tyr353. This, in turn, leads to an overload of Ca2+, which eventually interferes hepatic cells mitochondrial function in NAFLD [21]. Moreover, this phenomenon is not limited to the molecule that acquires the phosphate group itself, but may also occur in its downstream regulatory targets. For example, in nutrition repletion, the function of AMPK will be inhibited, preventing the addition of the phosphate group to downstream TBC1 domain family member 1 (TBC1D1). This results in the improved stability of downstream peroxisome proliferator-activated receptors (PPAR), thereby promoting the progression of NAFLD [22].

3.Affecting the localization of molecule in cell

Changes in phosphorylation states can impact the cellular localization of molecules. For example, Wilms' tumor 1-associating protein (WTAP) has been reported to be reduced in the liver cell nucleus under phosphorylation by tumor necrosis factor alpha (TNFα) in nonalcoholic steatohepatitis (NASH) condition [23]. In addition, the phosphorylated form of fork head box protein (FOX) can be translocated from the nucleus to the cytoplasm under certain conditions. This translocation leads to decreased expression of downstream targeted genes due to reduced transcriptional activity in the nucleus, thereby exacerbating NAFLD [24, 25].

4.Transforming molecular function

Phosphorylation modification often serves as a marker for transforming the function of a molecule. Acetyl-CoA carboxylase (ACC) catalyzes the transformation of acetyl-CoA to malonyl-CoA. As a substrate, malonyl-CoA can improve fatty acid oxidation by allosterically inhibiting carnitine O-palmitoyl transferase 1 (CPT1) [26]. Therefore, acetyl-CoA carboxylase (ACC) is essential for regulating glycolipid metabolism and the tricarboxylic acid cycle to maintain normal metabolic activity. Numerous studies have shown that an increase in ACC content in hepatocytes may induce NAFLD and NASH [27-31], which further enhances the possibility of conversion to HCC [32]. However, the phosphorylation of ACC can reverse its original harmful effects and subsequently improve NAFLD and NASH [27, 32-35]. Similar findings can be seen for other molecules ,such as sterol regulatory element-binding protein (SREBP)[27, 29, 31, 33, 36-38] and eukaryotic initiation factor 2B (eIF2B)[39, 40], which regulate in lipid metabolism and endoplasmic reticulum stress, respectively. Phosphorylated and non-phosphorylated forms of the same molecule can be viewed as a regulatory switch governing their respective enzymatic activities, with one state contributing to disease pathology while the other state may mitigate it.

Additionally, interplay among signaling pathways enables phosphate groups to modulate the functional interconversion between different pathways and molecules. Silybin is commonly utilized in NASH, where the activated JNK via phosphorylation is involved in inflammation. Silybin has the capacity to transfer the phosphate group from JNK to Insulin receptor substrate 1 (IRS1), and the subsequently, IRS1 bearing phosphate group can counteract insulin resistance to ameliorate NASH[41]. In the presence of Silybin, the phosphorylation of IRS1 can modulate its activity, leading to potential amelioration of NAFLD.

5.Cooperating with other protein modification

Phosphorylation represents just one facet of protein modification, frequently triggering alterations in conjunction with other groups, thereby fostering interplay between them. Ubiquitination assumes a critical function in protein degradation and governs numerous fundamental processes, including cell division, fate determination, and migration, often exhibiting correlation with phosphorylation [42]. For instance, the attachment of the phosphate group has been documented to alter the transcription of apoptosis signal-regulating kinase 1 (ASK1), recruiting ubiquitination at its 3' end, thereby activating ASK1 to facilitate the progression of NAFLD [43]. Acetylation is widely acknowledged as a common mechanism for regulating molecular transcription, primarily involved in the maintenance of cellular energy balance, regulation of gene expression, and modulation of metabolic pathways [44]. In instances of inadequate nutrition, the phosphorylated histone acetyltransferase Tip60 facilitates the acetylation process, leading to the disruption of autophagy in NAFLD [45]. Furthermore, the process of phosphorylating the oxysterol receptor α (LXRα) at the S196A site has been found to regulate hepatic chromatin acetylation, thereby decreasing the likelihood of developing hepatic inflammation and fibrosis [46]. Phosphorylation is not only associated with ubiquitination and acetylation, but also has been linked to methylation [47] and glycosylation [31]. The process of protein modification involves intricate interactions between different types of modifications. To fully comprehend the pathological mechanism of SLD, it is essential to examine the interplay among diverse protein modifications in a comprehensive manner.

Signaling pathways for regulating phosphorylation in SLD

Published research on phosphorylation in simple liver disease (SLD) has primarily concentrated on the phosphorylated AMPK, AKT, and NF-kB, as depicted in **Figure 1**. Altered levels of molecular phosphorylation have been linked to various downstream effects, including adipogenesis, steatosis, inflammatory responses, oxidative stress, fibrosis, insulin resistance, autophagy, and mitochondrial dysfunction[8, 9, 48]. While these three pathways have been extensively investigated, they are associated with different functions for SLD. AMPK is primarily involved in regulating abnormal fat metabolism in SLD, AKT is mainly associated with insulin resistance and abnormal glucose metabolism, and NF-kB is closely linked to inflammatory responses and immune abnormalities. The phosphorylation modifications mediated by these pathways may ultimately interact to contribute to SLD, and will be further discussed in the subsequent sections.

1.AMPK

The AMPK pathway is of great value in detecting energy status in eukaryotic cells, initiating energy insufficiency, and thus contributing to the process of cellular metabolism and energy transformation [49]. In Homo sapiens, AMPK predominantly occurs as heterotrimers, comprising one catalytic subunit α and another two regulatory subunits β and γ [50]. Additionally, subunitα exhibits catalytic activity, featuring an activation loop motif in close proximity to its ATP binding site. Typically, kinase domains are rendered active solely when the conserved threonine residues within the activation loop are phosphorylated, serving as a common indicator of AMPK activation [51]. Another area abundant in serine and threonine, known as the ST loop, exists. Certain residues within the ST loop have the potential to undergo phosphorylation under particular conditions, leading to their binding to the kinase domain and creating steric hindrance at the Thr172 site. This, in turn, hinders the activation of AMPK [52].

The association between AMPK and SLD is intimate. In response to external stimuli, phosphorylation of the Thr172 site on subunitα can be induced, leading to enhanced de novo lipogenesis and mitigation of SLD. Triptolide [53]、ginsenoside Rg1 [54]、green tea polyphenols [55]、alogliptin [56] and aerobic exercise [17] have currently been reported that they can participate in regulating the phosphorylation of AMPK at Thr172 site, effectively improving SLD. Furthermore, certain kinases, including liver kinase B1 (LKB1), TAK1, and the tripartite motif-containing protein (TRIM) family, as well as the fibroblast growth factor (FGF) family, have the capacity to modulate the phosphorylation of AMPK at the Thr172 site. This thus leads to the regulation in downstream signaling molecules, such as ACC and SREBP, primarily involving in de novo lipid synthesis and abnormal fatty accumulation. Currently, there is a dearth of research on the association between AMPK regulatory subunits and SLD. Further investigation is warranted to ascertain whether the regulatory subunits are implicated in the stability of AMPK phosphorylation conformation.

2. AKT

Phosphatidylinositol 3-kinase 3 (PI3K) is a dimer consisting of one subunit known as p85 and one catalytic subunit referred to as p110. Its primary function involves modifying the protein structure of AKT, thereby activating or inhibiting downstream substrates through phosphorylation. This process plays a crucial role in regulating cellular life processes, such as proliferation, differentiation, apoptosis, migration and others.

Upon receiving phosphorylation groups from activated PI3K, AKT typically facilitates the

regulation of downstream signaling pathways. For insulin resistance in skeletal muscle, AKT has been documented to primarily exert its effects on the mammalian target of rapamycin (mTOR) [57, 58] and FOX families [24, 25] to promote insulin resistance; Simultaneously, the excessive release of insulin molecules return to active the phosphorylation of IRS, which subsequently triggers the phosphorylation of AKT [59-61], creating a self-perpetuating cycle. Furthermore, the phosphorylation of AKT can induce irregularities in cell cycle proteins, thereby contributing to cell apoptosis and influencing SLD [62].

In contrast to AMPK, which predominantly controls the synthesis of lipids and breakdown of molecules, the activation of the phosphorylated AKT are primarily associated with insulin resistance and apoptosis in SLD.

3. NF-kB

NF-kB proteins typically form heterodimeric complexes with p65 and p50, which are rendered inactive in the cytoplasm due to their association with the NF-kB inhibitor epsilon (IkB). Upon activation of upstream signaling factors, IkB is phosphorylated by IkB kinase, leading to its dissociation from the trimer [63]. Consequently, NF-kB is able to expose its nuclear localization sequence (NLS), facilitating its rapid translocation from the cytoplasm to the nucleus binding with specific DNA sequences, thereby promoting the expression of downstream molecule. Literature suggests that the NF-kB primarily causes the activation of inflammation and immune dysregulation in SLD.

The identification of the phosphorylation of TAK1 [26, 64-68] and focal adhesion kinase (FAK) [69] has been documented to enhance the function of a range of inflammatory cytokines, such as TNFα, transforming growth factor β (TGFβ), and interleukin family, leading to the activation of NF-kB, inducing inflammatory reactions and immune irregularities. Furthermore, in research investigating the use of metformin to mitigate SLD [70, 71], it has been attributed to the ability of metformin to stimulate the phosphorylation of AMPK, thereby reducing subunit p65 and suppressing the NF-kB inflammatory response.

Therapeutic progress targeted in phosphorylation of SLD

Owing to the absence of specific drugs for SLD, current mainstream treatment approaches primarily involve lifestyle modifications and weight reduction. Nevertheless, these interventions may not yield favorable outcomes for all individuals [72, 73]. Phosphorylation is a significant factor in the development of SLD, suggesting that therapies directed at phosphorylation processes could have a substantial impact on alleviating the condition. Consequently, this outlines the predominant treatment strategies and underlying fundamental mechanisms related to the phosphorylation of SLD.

1.Lifestyle intervention

The primary approach for managing SLD involves lifestyle intervention, which encompasses dietary modifications and physical activity. These interventions have been shown to impact the phosphorylation of SLD patients, indicating their potential as therapeutic targets. Further details are provided in **Table 2**.

Dietary modifications have the potential to regulate phosphorylation levels in NAFLD. Adjusting dietary composition and habits can yield significant effects in treatment or prevention of NAFLD, with the potential mechanisms primarily associated with AMPK phosphorylation. For instance, the consumption of beans [74] and tomatoes [75] has been shown to notably ameliorate

NAFLD by reducing body weight and inflammatory responses. Additionally, a blend of lard and soybean oil is recognized for its ability to lower cholesterol levels and shield the liver from inflammation [76]. Furthermore, the consumption of Ishige okamurae, an edible seaweed, may also offer beneficial support in the treatment and prevention of NAFLD [77]. Moreover, supplementing with a specific amount of carbon during pregnancy has been associated to reduced occurrence of NAFLD in offspring [78]. In terms of dietary habits, fasting is a crucial measure for alleviating and preventing NAFLD [47]. More specifically, alternate-day fasting is considered to hold significant value in enhancing the cognitive function of NAFLD patients by reducing oxidative stress and mitigating microglial over-activation in the central nervous system [79].

Physical activity potentially reduce fatty accumulation and inflammation in the liver, making it a viable strategy for the treatment of NAFLD and NASH [73]. Additionally, exercise has been shown to ameliorate metabolic abnormalities such as insulin resistance and hypertriglyceridemia to some extent [80, 81]. Evidence from liver biopsy confirms that exercise can mitigate or improve hepatitis in NASH patients [82]. The primary mechanism through which exercise exerts these benefits is by increasing phosphorylated AMPK, thereby inhibiting genes associated with metabolic disorders and fatty accumulation.

2.Prescriptions

While there are no authorized pharmaceutical drugs for NAFLD and NASH, various prescription medications have shown promising results in clinical settings, particularly those targeting phosphorylation regulation. **Table 3** provides an overview of the advancements in utilizing phosphorylation-regulating prescriptions for the management in NAFLD and NASH.

The current treatment for NAFLD with pharmaceutical interventions primarily focuses on hypoglycemic medications, with metformin and sodium-dependent glucose transporters 2 inhibitors (SGLT-2i) being the most studied. Metformin is frequently utilized in clinical practice for patients with NAFLD and comorbid obesity and abnormal glucose metabolism due to its favorable effects on weight reduction and blood sugar levels [44, 83]. Its mechanism of action in NAFLD involves phosphorylation regulation targeting AMPK and glycogen synthase kinase 3β (GSK-3β), leading to macrophage polarization, reduction in inflammatory cytokines, and improvement in glycolipid metabolism and weight reduction [70, 71]. Additionally, metformin can ameliorate insulin resistance by regulating the phosphorylated ACC family, thereby mitigating NAFLD and NASH [84]. SGLT-2 inhibitors, a novel class of hypoglycemic drugs, function by reducing sugar absorption in the kidneys and increasing urine sugar excretion [85], thereby improving cardiovascular and cerebrovascular health, promoting weight loss, and normalizing metabolism, especially in NAFLD patients with diabetes [86, 87]. The phosphorylation regulation of SGLT2i primarily involves AMPK, particularly in the case of Empagliflozin[88, 89] and Dapagliflozin[28]. These drugs have been reported to enhance downstream targeted molecules by promoting their phosphorylation, thereby reducing fatty accumulation and inhibiting the release of inflammatory cytokines through pathways such as mTOR or NF-kB. However, there is vague on the regulation of glucagon-like peptide (GLP) receptor agonists on the phosphorylation in SLD, likely due to challenges related to dosing inconvenience, heterogeneity, and diversity of types.

Additionally, medications with hepatoprotective properties such as silybin and ursodeoxycholic acid (UDCA) are integral in the regulation of phosphorylation in NAFLD and are commonly utilized in the treatment of NASH [29, 41, 90, 91]. Silybin, a widely used hepatoprotective drug, demonstrates efficacy in treating elevated levels of glutamic-pyruvic

transaminase or glutamic oxaloacetic transaminase. Studies have indicated that silybin can ameliorate NAFLD by modulating caspase 8 and fatty acid synthase-associated protein, thereby improving insulin resistance, mitigating inflammation through inhibition of JNK phosphorylation [38, 41]. UDCA exhibits anti-inflammatory and antioxidant properties, effectively improving mitochondrial dysfunction, particularly under obesity-related conditions, and is suitable for patients with biliary obstruction. Clinical trials have shown that UDCA enhances energy expenditure in hepatic cells, promotes mitochondrial biogenesis, and improves bile acid metabolism by inhibiting NF-kB and signal transducer and activator of transcription 3 (STAT3) phosphorylation, rendering it an effective treatment for NAFLD [90, 91].

Furthermore, aspirin, a typical anti-inflammatory drug, possesses antipyretic, analgesic, and anti-rheumatic properties that have been found to alleviate NAFLD by modulating the AMPK phosphorylation. Aspirin appears to mitigate NAFLD by decreasing lipid biosynthesis and inflammation, thereby promoting catabolic metabolism through the activation of PPARδ and peroxisome proliferator-activated receptor gamma coactivator 1-alpha (PGC-1α) [92]. In addition, feniconazole nitrate has been identified as having potential in regulating phosphorylation changes with NAFLD and has been reported to alleviate the condition by activating facilitated glucose transporter member 4 (GLUT4) via the promotion of AKT phosphorylation at the Ser473 site and by blocking PPARγ phosphorylation at the Ser273 site mediated by cyclin-dependent kinases 5 (CDK5), thereby increasing the expression of adipogenic genes such as ACC. [93]. Although aspirin and feniconazole nitrate are not common in the treatment of NAFLD, their pharmacological mechanisms involve the PPAR pathway, showing crucial function in regulating inflammation, insulin resistance, abnormal fat metabolism and others.

3.Traditional Chinese medicine

In recent times, Chinese herbal medicine has demonstrated distinctive efficacy in the management of chronic ailments, particularly in the context of SLD. The enigmatic therapeutic properties and interplay of traditional Chinese medicine are increasingly recognized for their significance. Current investigations into traditional Chinese medicine encompass the study of Chinese medicine monomers, Chinese medicine prescriptions, and the active constituents of Chinese medicine, revealing close associations between their efficacy and the modulation of phosphorylation.

In the context of regulating phosphorylation, over 20 traditional Chinese medicines or active components of traditional Chinese medicine have been considered in treating NAFLD or NASH, including: breviscapine [94], anthocyanin [95], coffeeberry [96], cordycepin [97], salidroside [98], resveratrol [37, 99], triptolide [34], berberine [100, 101], morin [102], corosolic acid [103, 104], ginsenoside [105, 106], vine tea polyphenol [107], quercetin [108], aurantio-obtusin [109], patchouli alcohol [110], zingerone [111], scopoletin/umbelliferone [112], astragalus mongholicus polysaccharides [113], lycopus lucidus Turcz. ex Bent [114], gentiana scabra [115], artemisia capillaris [116], mogrosides [117] and Fufang Zhenzhu Tiaozhi formula [118]. The phosphorylation sites and associated regulatory mechanisms of these substances are summarized in **Table 4**.

The research methodologies and potential regulatory mechanisms of traditional Chinese medicine through phosphorylation appear to align with traditional Chinese medicine theories to some extent. For instance, Coptis chinensis, containing berberine, demonstrates efficacy in improving SLD by reducing blood sugar and fat levels, exhibiting antioxidant properties, and

mitigating inflammatory reactions [100, 101]. Berberine's ability to enhance the phosphorylation of various signaling molecules, including IRS, AKT, AMPK, and JNK, contributes to reducing insulin resistance, ameliorating inflammatory responses, alleviating oxidative stress, and diminishing lipid formation [100, 101]. The regulation of phosphorylation may provide a plausible rationale for the diverse effects of individual traditional Chinese medicines or their constituents. While further validation is necessary, this implies that the regulation of phosphorylation holds significant potential in treatment of SLD by traditional Chinese medicine.

4.Others

There are also some new findings that are important in regulating the phosphorylation of NAFLD and NASH, mainly including medical materials and chemical compounds. A new type of nanoparticle loaded with nifedipine can promote autophagy and reduce liver fat, where it enhances water solubility without modifying the chemical structure while allows prolonged release in vivo. Therefore, by increasing autophagic clearance through Ca2+/calmodulin-dependent kinase II phosphorylation, this nanoparticle leads to suppression of metabolic derangements associated in NAFLD [119]. Additionally, a hepatic-targeted delivery system utilizing oxidized starch-lysozyme nanocarriers to administer resveratrol has been shown to elevate p-AMPK and p-IRS, thereby reducing adipogenesis and insulin resistance [37]. This system achieves precise liver targeting by employing covalently conjugated galactose, recognized by the asialoglycoprotein receptors which is specifically expressed in hepatocytes, and ultimately facilitating the delivery of drugs to modulate phosphorylation.

Furthermore, there have been recent discoveries of newly activated molecules, or synthetic chemicals, that exhibit potential therapeutic properties and are being investigated as potential target for NAFLD and NASH. For instance, SYSU-3d has been found to activate the phosphorylation of heat shock factor 1 (HSF1), thereby promoting PGC-1a to inhibit oxidative stress and inflammation [119]. AdipoRon, the first small molecule adipoR agonist, particularly its subtype Q7, is thought to alleviate NAFLD by enhancing the phosphorylation of AMPK [120]. Additionally, a novel liver-specific ACC inhibitor known as ND-654 mimics the function of ACC phosphorylation and hinders the progression of liver de novo lipogenesis and hepatocellular carcinoma [32]. Moreover, an unexplored type IV collagen inhibitor, Cpd17, influences the phosphorylation of the ATX-LPA axis and holds significant potential in treating NAFLD [121].

Challenges and prospects

As SLD continues to rise, there is a growing global focus on the prevention and management of SLD. However, the precise mechanism of SLD remains unclear, and there is currently no specific pharmaceutical intervention targeting SLD. Proteins play a direct role as downstream molecules in exerting functional effects. Protein modification can directly influence the structure or function of proteins, with phosphorylation being the most extensively studied form of modification. Abnormal regulation of phosphorylation at different amino acid residues and their specific sites can significantly impact the development of SLD. Therefore, investigating the role of phosphorylation in the fundamental nature of SLD is of great importance. Nevertheless, based on current research, the following areas can provide a framework for future research on phosphorylation-related mechanisms in SLD.

1. Utilizing a combination of multiple omics methodologies and single-cell technology is essential for a comprehensive exploration of phosphorylation.

Current research on phosphorylation has been predominantly focused on the effects of specific molecules or phosphorylation sites, thereby elucidating their regulatory role in signaling pathways or phenotypic outcomes. For instance, extensive studies have been conducted on the phosphorylation of AMPK at Thr172, revealing its regulation by various factors and its impact on the development of SLD [22, 27, 29, 32, 35, 38, 122-124].

There is currently no specific elucidation of the involvement of upstream kinases in the regulation of phosphorylation, the influence of phosphorylation modification on protein structure or function, and the validation of novel phosphorylation sites. The emergence of bioinformatics technology has provided opportunities to investigate whether changes in protein-level phosphorylation are implicated in regulating other molecules at the transcriptomic level or in the modulation of protein-protein interactions through high-throughput multi-omics analysis. Furthermore, the examination of potential disparities in the phosphorylation modification of the same protein across different cell types and its impact on various cellular functions or fates, in conjunction with single-cell mass spectrometry technology, may yield insights. For instance, the emerging technology of Cytometry by Time of Flight utilizes metal ions to categorize cell subpopulations for high-throughput exploration of distinct intracellular proteomics and modification sites [125].

2.Inflammatory signaling pathway specific phosphorylase inhibitors.

In the development of Specific Learning Disorders (SLD), inflammation and various immune irregularities are fundamental mechanisms, with phosphorylation frequently assuming a central facilitative role, including activation of the NF-kB pathway, JNK pathway, AKT pathway, and others. Currently, while there is a dearth of specific pharmaceutical interventions directly targeting SLD, certain medications have demonstrated the ability to suppress inflammatory signaling pathways and cytokine phosphorylation, thereby mitigating the progression of SLD, such as Silybin [41] and UDCA [91]. Nevertheless, these medications do not selectively inhibit the phosphorylation of inflammation-related signaling pathways and lack substantial evidence-based support, necessitating further investigation.

3.Phosphorylation regulation in insulin resistance in SLD.

The occurrence of insulin resistance can induce metabolic disorders, further inducing inflammatory reactions and immune abnormalities [126]. At the same time, insulin resistance is regarded as to be closely related to a decrease in muscle and bone content [127, 128], which in turn induces or exacerbates the occurrence of SLD. Although the mechanism research is not yet clear, the regulation of phosphorylation is regarded to be widely involved, mainly through PI3K-AKT signaling and IRS mediated insulin resistance [40, 129, 130]. Inhibiting the phosphorylation of corresponding proteins or targeting AMPK phosphorylation at Th172 through kinase has the potential to reverse insulin resistance, but further research is needed in the future.

4.Uncoupling protein (UCP) and SLD

UCP may show enormous potential in the treatment of SLD under oxidative phosphorylation. UCP have specific physiological functions, and hibernating and newborn animals can use uncoupling proteins to convert some of the energy originally used for ATP production into heat [131]. On the one hand, the genotype of UCP can be associated with patient prognosis. It is reported that UCP1 (AG+GG) genotype is positively correlated with the severity of hepatic steatosis [132]. On the other hand, UCP has the potential targeting phosphorylation to improve SLD. Although there is no approved drug for SLD, there are many drugs reckoned as good

candidates via phosphorylation[133]. For example, thyroxine can promote the expression of UCP, which allows more to join the uncoupling process, thereby increasing heat production and oxygen consumption[134]. Besides, thyroid hormone can increase the number of sodium and potassium pumps on the cell membrane, leading to more ATP consumption and promoting the process of oxidative phosphorylation [134]. At present, the one of new pharmacology in clinical trial for NAFLD is thyroid hormone receptor β agonists targeting to liver, significantly influencing UCP and then reducing liver fatty accumulation and improve NASH [135], which show great potential for future exploration.

Conclusion

The progress of phosphorylation is of great value and shares close association with SLD, whether in pathogenesis or treatment. It is indicated that phosphorylation mainly affects SLD, where AMPK, AKT, and NF-kB are key factors closely related to de novo lipogenesis, metabolic disorders, inflammatory reactions, and abnormal immunity. In terms of treatment, although there are no approved drugs that can treat SLD, many potential drugs that can alleviate SLD through phosphorylation. Further exploration of the mechanism of phosphorylation in SLD can benefit significantly clinical. In addition, more detailed research is necessary for studying phosphorylation in SLD, especially combining multi omics and single-cell technology to accurately explore the pathogenesis of SLD. In all, phosphorylation is of great value as a pathogenesis and therapeutic target for SLD.

Reference:

1. Rinella ME, Lazarus JV, Ratziu V, Francque SM, Sanyal AJ, Kanwal F, Romero D, Abdelmalek MF, Anstee QM, Arab JP, et al: **A multisociety Delphi consensus statement on new fatty liver disease nomenclature.** *Journal of Hepatology* 2023, **79:**1542.
2. Arora U, Biswas S, Aggarwal S, Duseja A, Shalimar: **MASLD screening and diagnostic algorithms are interchangeable from existing NAFLD literature.** *Journal of Hepatology* 2023.
3. Hagström H, Vessby J, Ekstedt M, Shang Y: **99% of patients with NAFLD meet MASLD criteria and natural history is therefore identical.** *Journal of Hepatology* 2023.
4. Yang A, Zhu X, Zhang L, Ding Y: **Transitioning from NAFLD to MAFLD and MASLD: Consistent prevalence and risk factors in a Chinese cohort.** *Journal of Hepatology* 2023.
5. Perazzo H, Pacheco AG, Griep RH: **Changing from NAFLD through MAFLD to MASLD: Similar prevalence and risk factors in a large Brazilian cohort.** *Journal of Hepatology* 2023.
6. Younossi ZM, Koenig AB, Abdelatif D, Fazel Y, Henry L, Wymer M: **Global epidemiology of nonalcoholic fatty liver disease-Meta-analytic assessment of prevalence, incidence, and outcomes.** *Hepatology (Baltimore, Md)* 2016, **64:**73.
7. Anstee QM, Reeves HL, Kotsiliti E, Govaere O, Heikenwalder M: **From NASH to HCC: current concepts and future challenges.** *Nature Reviews Gastroenterology & Hepatology* 2019, **16:**411.
8. Powell EE, Wong VW, Rinella M: **Non-alcoholic fatty liver disease.** *Lancet* 2021, **397:**2212.
9. Wang XJ, Malhi H: **Nonalcoholic Fatty Liver Disease.** *Ann Intern Med* 2018, **169:**ITC65.
10. Luo F, Smagris E, Martin SA, Vale G, McDonald JG, Fletcher JA, Burgess SC, Hobbs HH, Cohen JC: **Hepatic TM6SF2 Is Required for Lipidation of VLDL in a Pre-Golgi Compartment in Mice and Rats.** *Cellular and Molecular Gastroenterology and Hepatology* 2022, **13:**879.
11. Luo F, Oldoni F, Das A: **TM6SF2: A Novel Genetic Player in Nonalcoholic Fatty Liver and Cardiovascular Disease.** *Hepatology Communications* 2022, **6:**448.
12. Bilbrough T, Piemontese E, Seitz O: **Dissecting the role of protein phosphorylation: a chemical biology toolbox.** *Chem Soc Rev* 2022, **51:**5691.
13. Ullah R, Rauf N, Nabi G, Ullah H, Shen Y, Zhou Y-D, Fu J: **Role of Nutrition in the Pathogenesis and Prevention of Non-alcoholic Fatty Liver Disease: Recent Updates.** *International Journal of Biological Sciences* 2019, **15:**265.
14. Bonnet L, Alexandersson I, Baboota RK, Kroon T, Oscarsson J, Smith U, Boucher J: **Cellular senescence in hepatocytes contributes to metabolic disturbances in NASH.** *Frontiers In Endocrinology* 2022, **13:**957616.
15. Chen B, Sun L, Zeng G, Shen Z, Wang K, Yin L, Xu F, Wang P, Ding Y, Nie Q, et al: **Gut bacteria alleviate smoking-related NASH by degrading gut nicotine.** *Nature* 2022, **610:**562.
16. Koh Y-C, Lin Y-C, Lee P-S, Lu T-J, Lin K-Y, Pan M-H: **A multi-targeting strategy to ameliorate high-fat-diet- and fructose-induced (western diet-induced) non-alcoholic fatty liver disease (NAFLD) with supplementation of a mixture of legume ethanol extracts.** *Food & Function* 2020, **11:**7545.
17. Diniz TA, de Lima Junior EA, Teixeira AA, Biondo LA, da Rocha LAF, Valadão IC, Silveira LS, Cabral-Santos C, de Souza CO, Rosa Neto JC: **Aerobic training improves NAFLD markers and insulin resistance through AMPK-PPAR-α signaling in obese mice.** *Life Sciences* 2021, **266:**118868.

18. Tilg H, Moschen AR, Roden M: **NAFLD and diabetes mellitus.** *Nature Reviews Gastroenterology & Hepatology* 2017, **14:**32.
19. Adams LA, Anstee QM, Tilg H, Targher G: **Non-alcoholic fatty liver disease and its relationship with cardiovascular disease and other extrahepatic diseases.** *Gut* 2017, **66:**1138.
20. Chen B, Sun L, Zeng G, Shen Z, Wang K, Yin L, Xu F, Wang P, Ding Y, Nie Q, et al: **Gut bacteria alleviate smoking-related NASH by degrading gut nicotine.** *Nature* 2022, **610:**562.
21. Yu T, Zheng E, Li Y, Li Y, Xia J, Ding Q, Hou Z, Ruan XZ, Zhao L, Chen Y: **Src-mediated Tyr353 phosphorylation of IP3R1 promotes its stability and causes apoptosis in palmitic acid-treated hepatocytes.** *Exp Cell Res* 2021, **399:**112438.
22. Chen ZY, Sun YT, Wang ZM, Hong J, Xu M, Zhang FT, Zhou XQ, Rong P, Wang Q, Wang HY, et al: **Rab2A regulates the progression of nonalcoholic fatty liver disease downstream of AMPK-TBC1D1 axis by stabilizing PPARgamma.** *PLoS Biol* 2022, **20:**e3001522.
23. Li X, Ding K, Li X, Yuan B, Wang Y, Yao Z, Wang S, Huang H, Xu B, Xie L, et al: **Deficiency of WTAP in hepatocytes induces lipoatrophy and non-alcoholic steatohepatitis (NASH).** *Nature Communications* 2022, **13:**4549.
24. Liu XL, Pan Q, Cao HX, Xin FZ, Zhao ZH, Yang RX, Zeng J, Zhou H, Fan JG: **Lipotoxic Hepatocyte-Derived Exosomal MicroRNA 192-5p Activates Macrophages Through Rictor/Akt/Forkhead Box Transcription Factor O1 Signaling in Nonalcoholic Fatty Liver Disease.** *Hepatology* 2020, **72:**454.
25. Ma J, Hu W, Zhang D, Xie J, Duan C, Liu Y, Wang Y, Xu X, Cheng K, Jin B, et al: **CD226 knockout alleviates high-fat diet induced obesity by suppressing proinflammatory macrophage phenotype.** *J Transl Med* 2021, **19:**477.
26. McGarry JD, Leatherman GF, Foster DW: **Carnitine palmitoyltransferase I. The site of inhibition of hepatic fatty acid oxidation by malonyl-CoA.** *Journal of Biological Chemistry* 1978, **253:**4128.
27. Li R, Li Y, Yang X, Hu Y, Yu H, Li Y: **Reducing VEGFB accelerates NAFLD and insulin resistance in mice via inhibiting AMPK signaling pathway.** *J Transl Med* 2022, **20:**341.
28. Li L, Li Q, Huang W, Han Y, Tan H, An M, Xiang Q, Zhou R, Yang L, Cheng Y: **Dapagliflozin Alleviates Hepatic Steatosis by Restoring Autophagy via the AMPK-mTOR Pathway.** *Front Pharmacol* 2021, **12:**589273.
29. Liu X, Hu M, Ye C, Liao L, Ding C, Sun L, Liang J, Chen Y: **Isosilybin regulates lipogenesis and fatty acid oxidation via the AMPK/SREBP-1c/PPARalpha pathway.** *Chem Biol Interact* 2022, **368:**110250.
30. Ma P, Huang R, Jiang J, Ding Y, Li T, Ou Y: **Potential use of C-phycocyanin in non-alcoholic fatty liver disease.** *Biochem Biophys Res Commun* 2020, **526:**906.
31. Zhao N, Zhang X, Ding J, Pan Q, Zheng MH, Liu WY, Luo G, Qu J, Li M, Li L, et al: **SEMA7AR148W mutation promotes lipid accumulation and NAFLD progression via increased localization on the hepatocyte surface.** *JCI Insight* 2022, **7**.
32. Lally JSV, Ghoshal S, DePeralta DK, Moaven O, Wei L, Masia R, Erstad DJ, Fujiwara N, Leong V, Houde VP, et al: **Inhibition of Acetyl-CoA Carboxylase by Phosphorylation or the Inhibitor ND-654 Suppresses Lipogenesis and Hepatocellular Carcinoma.** *Cell Metab* 2019, **29:**174.
33. Zhang Q, Yu K, Cao Y, Luo Y, Liu Y, Zhao C: **miR-125b promotes the NF-kappaB-mediated inflammatory response in NAFLD via directly targeting TNFAIP3.** *Life Sci* 2021, **270:**119071.

34. Huang R, Guo F, Li Y, Liang Y, Li G, Fu P, Ma L: **Activation of AMPK by triptolide alleviates nonalcoholic fatty liver disease by improving hepatic lipid metabolism, inflammation and fibrosis.** *Phytomedicine* 2021, **92:**153739.
35. Chen Y, He X, Chen X, Li Y, Ke Y: **SeP is elevated in NAFLD and participates in NAFLD pathogenesis through AMPK/ACC pathway.** *J Cell Physiol* 2021, **236:**3800.
36. Moreira GV, Araujo LCC, Murata GM, Matos SL, Carvalho CRO: **Kombucha tea improves glucose tolerance and reduces hepatic steatosis in obese mice.** *Biomed Pharmacother* 2022, **155:**113660.
37. Teng W, Zhao L, Yang S, Zhang C, Liu M, Luo J, Jin J, Zhang M, Bao C, Li D, et al: **The hepatic-targeted, resveratrol loaded nanoparticles for relief of high fat diet-induced nonalcoholic fatty liver disease.** *J Control Release* 2019, **307:**139.
38. Zhao ZH, Wang ZX, Zhou D, Han Y, Ma F, Hu Z, Xin FZ, Liu XL, Ren TY, Zhang F, et al: **Sodium Butyrate Supplementation Inhibits Hepatic Steatosis by Stimulating Liver Kinase B1 and Insulin-Induced Gene.** *Cell Mol Gastroenterol Hepatol* 2021, **12:**857.
39. Li J, Li X, Liu D, Zhang S, Tan N, Yokota H, Zhang P: **Phosphorylation of eIF2alpha signaling pathway attenuates obesity-induced non-alcoholic fatty liver disease in an ER stress and autophagy-dependent manner.** *Cell Death Dis* 2020, **11:**1069.
40. Qiao L, Men L, Yu S, Yao J, Li Y, Wang M, Yu Y, Wang N, Ran L, Wu Y, Du J: **Hepatic deficiency of selenoprotein S exacerbates hepatic steatosis and insulin resistance.** *Cell Death Dis* 2022, **13:**275.
41. Liu Y, Xu W, Zhai T, You J, Chen Y: **Silibinin ameliorates hepatic lipid accumulation and oxidative stress in mice with non-alcoholic steatohepatitis by regulating CFLAR-JNK pathway.** *Acta Pharm Sin B* 2019, **9:**745.
42. Rape M: **Ubiquitylation at the crossroads of development and disease.** *Nature Reviews Molecular Cell Biology* 2018, **19:**59.
43. Lan T, Hu Y, Hu F, Li H, Chen Y, Zhang J, Yu Y, Jiang S, Weng Q, Tian S, et al: **Hepatocyte glutathione S-transferase mu 2 prevents non-alcoholic steatohepatitis by suppressing ASK1 signaling.** *Journal of Hepatology* 2022, **76:**407.
44. Xing S, Poirier Y: **The protein acetylome and the regulation of metabolism.** *Trends Plant Sci* 2012, **17:**423.
45. Cheng X, Ma X, Zhu Q, Song D, Ding X, Li L, Jiang X, Wang X, Tian R, Su H, et al: **Pacer Is a Mediator of mTORC1 and GSK3-TIP60 Signaling in Regulation of Autophagosome Maturation and Lipid Metabolism.** *Mol Cell* 2019, **73:**788.
46. Becares N, Gage MC, Voisin M, Shrestha E, Martin-Gutierrez L, Liang N, Louie R, Pourcet B, Pello OM, Luong TV, et al: **Impaired LXRalpha Phosphorylation Attenuates Progression of Fatty Liver Disease.** *Cell Rep* 2019, **26:**984.
47. Byun S, Seok S, Kim YC, Zhang Y, Yau P, Iwamori N, Xu HE, Ma J, Kemper B, Kemper JK: **Fasting-induced FGF21 signaling activates hepatic autophagy and lipid degradation via JMJD3 histone demethylase.** *Nat Commun* 2020, **11:**807.
48. Anstee QM, Reeves HL, Kotsiliti E, Govaere O, Heikenwalder M: **From NASH to HCC: current concepts and future challenges.** *Nat Rev Gastroenterol Hepatol* 2019, **16:**411.
49. Hardie DG, Schaffer BE, Brunet A: **AMPK: An Energy-Sensing Pathway with Multiple Inputs and Outputs.** *Trends In Cell Biology* 2016, **26:**190.
50. Garcia D, Shaw RJ: **AMPK: Mechanisms of Cellular Energy Sensing and Restoration of**

Metabolic Balance. *Molecular Cell* 2017, **66:**789.

51. Hawley SA, Davison M, Woods A, Davies SP, Beri RK, Carling D, Hardie DG: **Characterization of the AMP-activated protein kinase kinase from rat liver and identification of threonine 172 as the major site at which it phosphorylates AMP-activated protein kinase.** *The Journal of Biological Chemistry* 1996, **271:**27879.
52. Hawley SA, Ross FA, Gowans GJ, Tibarewal P, Leslie NR, Hardie DG: **Phosphorylation by Akt within the ST loop of AMPK-α1 down-regulates its activation in tumour cells.** *The Biochemical Journal* 2014, **459:**275.
53. Huang R, Guo F, Li Y, Liang Y, Li G, Fu P, Ma L: **Activation of AMPK by triptolide alleviates nonalcoholic fatty liver disease by improving hepatic lipid metabolism, inflammation and fibrosis.** *Phytomedicine : International Journal of Phytotherapy and Phytopharmacology* 2021, **92:**153739.
54. Xiao Q, Zhang S, Yang C, Du R, Zhao J, Li J, Xu Y, Qin Y, Gao Y, Huang W: **Ginsenoside Rg1 Ameliorates Palmitic Acid-Induced Hepatic Steatosis and Inflammation in HepG2 Cells via the AMPK/NF-κB Pathway.** *International Journal of Endocrinology* 2019, **2019:**7514802.
55. Tan Y, Kim J, Cheng J, Ong M, Lao W-G, Jin X-L, Lin Y-G, Xiao L, Zhu X-Q, Qu X-Q: **Green tea polyphenols ameliorate non-alcoholic fatty liver disease through upregulating AMPK activation in high fat fed Zucker fatty rats.** *World Journal of Gastroenterology* 2017, **23:**3805.
56. Tobita H, Sato S, Yazaki T, Mishiro T, Ishimura N, Ishihara S, Kinoshita Y: **Alogliptin alleviates hepatic steatosis in a mouse model of nonalcoholic fatty liver disease by promoting CPT1a expression via Thr172 phosphorylation of AMPKα in the liver.** *Molecular Medicine Reports* 2018, **17:**6840.
57. Cheng X, Ma X, Zhu Q, Song D, Ding X, Li L, Jiang X, Wang X, Tian R, Su H, et al: **Pacer Is a Mediator of mTORC1 and GSK3-TIP60 Signaling in Regulation of Autophagosome Maturation and Lipid Metabolism.** *Molecular Cell* 2019, **73**.
58. Li L, Li Q, Huang W, Han Y, Tan H, An M, Xiang Q, Zhou R, Yang L, Cheng Y: **Dapagliflozin Alleviates Hepatic Steatosis by Restoring Autophagy via the AMPK-mTOR Pathway.** *Frontiers In Pharmacology* 2021, **12:**589273.
59. Qiao L, Men L, Yu S, Yao J, Li Y, Wang M, Yu Y, Wang N, Ran L, Wu Y, Du J: **Hepatic deficiency of selenoprotein S exacerbates hepatic steatosis and insulin resistance.** *Cell Death & Disease* 2022, **13:**275.
60. Xu M, Ge C, Zhu L, Qin Y, Du C, Lou D, Li Q, Hu L, Sun Y, Dai X, et al: **iRhom2 Promotes Hepatic Steatosis by Activating MAP3K7-Dependent Pathway.** *Hepatology (Baltimore, Md)* 2021, **73:**1346.
61. Wu H, Zhang T, Pan F, Steer CJ, Li Z, Chen X, Song G: **MicroRNA-206 prevents hepatosteatosis and hyperglycemia by facilitating insulin signaling and impairing lipogenesis.** *Journal of Hepatology* 2017, **66:**816.
62. Ma L, Lian Y, Tang J, Chen F, Gao H, Zhou Z, Hou N, Yi W: **Identification of the anti-fungal drug fenticonazole nitrate as a novel PPARγ-modulating ligand with good therapeutic index: Structure-based screening and biological validation.** *Pharmacological Research* 2021, **173:**105860.
63. Baldwin AS: **The NF-kappa B and I kappa B proteins: new discoveries and insights.** *Annual Review of Immunology* 1996, **14:**649.
64. Wang L, Zhang X, Lin ZB, Yang PJ, Xu H, Duan JL, Ruan B, Song P, Liu JJ, Yue ZS, et al: **Tripartite**

motif 16 ameliorates nonalcoholic steatohepatitis by promoting the degradation of phospho-TAK1. *Cell Metab* 2021, **33:**1372.

65. Wang J, Ma J, Nie H, Zhang XJ, Zhang P, She ZG, Li H, Ji YX, Cai J: **Hepatic Regulator of G Protein Signaling 5 Ameliorates Nonalcoholic Fatty Liver Disease by Suppressing Transforming Growth Factor Beta-Activated Kinase 1-c-Jun-N-Terminal Kinase/p38 Signaling.** *Hepatology* 2021, **73:**104.
66. Liu Y, Song J, Yang J, Zheng J, Yang L, Gao J, Tian S, Liu Z, Meng X, Wang JC, et al: **Tumor Necrosis Factor alpha-Induced Protein 8-Like 2 Alleviates Nonalcoholic Fatty Liver Disease Through Suppressing Transforming Growth Factor Beta-Activated Kinase 1 Activation.** *Hepatology* 2021, **74:**1300.
67. Huang C, Liu Q, Tang Q, Jing X, Wu T, Zhang J, Zhang G, Zhou J, Zhang Z, Zhao Y, et al: **Hepatocyte-specific deletion of Nlrp6 in mice exacerbates the development of non-alcoholic steatohepatitis.** *Free Radic Biol Med* 2021, **169:**110.
68. Qian Q, Li Y, Fu J, Leng D, Dong Z, Shi J, Shi H, Cao D, Cheng X, Hu Y, et al: **Switch-associated protein 70 protects against nonalcoholic fatty liver disease through suppression of TAK1.** *Hepatology* 2022, **75:**1507.
69. Ge C, Tan J, Dai X, Kuang Q, Zhong S, Lai L, Yi C, Sun Y, Luo J, Zhang C, et al: **Hepatocyte phosphatase DUSP22 mitigates NASH-HCC progression by targeting FAK.** *Nat Commun* 2022, **13:**5945.
70. Zamani-Garmsiri F, Hashemnia SMR, Shabani M, Bagherieh M, Emamgholipour S, Meshkani R: **Combination of metformin and genistein alleviates non-alcoholic fatty liver disease in high-fat diet-fed mice.** *J Nutr Biochem* 2021, **87:**108505.
71. Zamani-Garmsiri F, Ghasempour G, Aliabadi M, Hashemnia SMR, Emamgholipour S, Meshkani R: **Combination of metformin and chlorogenic acid attenuates hepatic steatosis and inflammation in high-fat diet fed mice.** *IUBMB Life* 2021, **73:**252.
72. Vilar Gomez E, Rodriguez De Miranda A, Gra Oramas B, Arus Soler E, Llanio Navarro R, Calzadilla Bertot L, Yasells Garcia A, Del Rosario Abreu Vazquez M: **Clinical trial: a nutritional supplement Viusid, in combination with diet and exercise, in patients with nonalcoholic fatty liver disease.** *Alimentary Pharmacology & Therapeutics* 2009, **30**.
73. Viveiros K: **The Role of Life Style Modifications in Comprehensive Non-Alcoholic Fatty Liver Disease Treatment.** *Clinical Liver Disease* 2021, **17:**11.
74. Koh YC, Lin YC, Lee PS, Lu TJ, Lin KY, Pan MH: **A multi-targeting strategy to ameliorate high-fat-diet- and fructose-induced (western diet-induced) non-alcoholic fatty liver disease (NAFLD) with supplementation of a mixture of legume ethanol extracts.** *Food Funct* 2020, **11:**7545.
75. Wu SJ, Huang WC, Yu MC, Chen YL, Shen SC, Yeh KW, Liou CJ: **Tomatidine ameliorates obesity-induced nonalcoholic fatty liver disease in mice.** *J Nutr Biochem* 2021, **91:**108602.
76. Yan S, Liu S, Qu J, Li X, Hu J, Zhang L, Liu X, Li X, Wang X, Wen L, Wang J: **A Lard and Soybean Oil Mixture Alleviates Low-Fat-High-Carbohydrate Diet-Induced Nonalcoholic Fatty Liver Disease in Mice.** *Nutrients* 2022, **14**.
77. Cha SH, Hwang Y, Heo SJ, Jun HS: **Diphlorethohydroxycarmalol Attenuates Palmitate-Induced Hepatic Lipogenesis and Inflammation.** *Mar Drugs* 2020, **18**.
78. Peng H, Xu H, Wu J, Li J, Wang X, Liu Z, Kim M, Jeon MS, Zhang KK, Xie L: **Maternal One-Carbon Supplement Reduced the Risk of Non-Alcoholic Fatty Liver Disease in Male**

Offspring. *Nutrients* 2022, **14**.

79. Zhang W, Wang J, Wang L, Shi R, Chu C, Shi Z, Liu P, Li Y, Liu X, Liu Z: **Alternate-day fasting prevents non-alcoholic fatty liver disease and working memory impairment in diet-induced obese mice.** *J Nutr Biochem* 2022, **110:**109146.
80. Diniz TA, de Lima Junior EA, Teixeira AA, Biondo LA, da Rocha LAF, Valadao IC, Silveira LS, Cabral-Santos C, de Souza CO, Rosa Neto JC: **Aerobic training improves NAFLD markers and insulin resistance through AMPK-PPAR-alpha signaling in obese mice.** *Life Sci* 2021, **266:**118868.
81. Gehrke N, Biedenbach J, Huber Y, Straub BK, Galle PR, Simon P, Schattenberg JM: **Voluntary exercise in mice fed an obesogenic diet alters the hepatic immune phenotype and improves metabolic parameters - an animal model of life style intervention in NAFLD.** *Sci Rep* 2019, **9:**4007.
82. Stine JG, Xu D, Schmitz K, Sciamanna C, Kimball SR: **Exercise Attenuates Ribosomal Protein Six Phosphorylation in Fatty Liver Disease.** *Dig Dis Sci* 2020, **65:**3238.
83. Marchesini G, Bianchi G, Tomassetti S, Zoli M, Melchionda N: **Metformin in non-alcoholic steatohepatitis.** *The Lancet* 2001, **358:**893.
84. Greenhill C: **Metformin, weight loss and NAFLD.** *Nat Rev Endocrinol* 2010, **6:**296.
85. Malaga G, Ruiz EF: **SGLT-2 inhibitors for people with type 2 diabetes.** *Lancet* 2019, **394:**559.
86. Townsend SA, Newsome PN: **Review article: new treatments in non-alcoholic fatty liver disease.** *Aliment Pharmacol Ther* 2017, **46:**494.
87. Androutsakos T, Nasiri-Ansari N, Bakasis AD, Kyrou I, Efstathopoulos E, Randeva HS, Kassi E: **SGLT-2 Inhibitors in NAFLD: Expanding Their Role beyond Diabetes and Cardioprotection.** *Int J Mol Sci* 2022, **23**.
88. Kuchay MS, Krishan S, Mishra SK, Farooqui KJ, Singh MK, Wasir JS, Bansal B, Kaur P, Jevalikar G, Gill HK, et al: **Effect of Empagliflozin on Liver Fat in Patients With Type 2 Diabetes and Nonalcoholic Fatty Liver Disease: A Randomized Controlled Trial (E-LIFT Trial).** *Diabetes Care* 2018, **41:**1801.
89. Nasiri-Ansari N, Nikolopoulou C, Papoutsi K, Kyrou I, Mantzoros CS, Kyriakopoulos G, Chatzigeorgiou A, Kalotychou V, Randeva MS, Chatha K, et al: **Empagliflozin Attenuates Non-Alcoholic Fatty Liver Disease (NAFLD) in High Fat Diet Fed ApoE((-/-)) Mice by Activating Autophagy and Reducing ER Stress and Apoptosis.** *Int J Mol Sci* 2021, **22**.
90. Traussnigg S, Schattenberg JM, Demir M, Wiegand J, Geier A, Teuber G, Hofmann WP, Kremer AE, Spreda F, Kluwe J, et al: **Norursodeoxycholic acid versus placebo in the treatment of non-alcoholic fatty liver disease: a double-blind, randomised, placebo-controlled, phase 2 dose-finding trial.** *Lancet Gastroenterol Hepatol* 2019, **4:**781.
91. Chen YS, Liu HM, Lee TY: **Ursodeoxycholic Acid Regulates Hepatic Energy Homeostasis and White Adipose Tissue Macrophages Polarization in Leptin-Deficiency Obese Mice.** *Cells* 2019, **8**.
92. Han YM, Lee YJ, Jang YN, Kim HM, Seo HS, Jung TW, Jeong JH: **Aspirin Improves Nonalcoholic Fatty Liver Disease and Atherosclerosis through Regulation of the PPARdelta-AMPK-PGC-1alpha Pathway in Dyslipidemic Conditions.** *Biomed Res Int* 2020, **2020:**7806860.
93. Ma L, Lian Y, Tang J, Chen F, Gao H, Zhou Z, Hou N, Yi W: **Identification of the anti-fungal drug fenticonazole nitrate as a novel PPARgamma-modulating ligand with good therapeutic**

index: Structure-based screening and biological validation. *Pharmacol Res* 2021, **173:**105860.

94. Lan T, Jiang S, Zhang J, Weng Q, Yu Y, Li H, Tian S, Ding X, Hu S, Yang Y, et al: **Breviscapine alleviates NASH by inhibiting TGF-beta-activated kinase 1-dependent signaling.** *Hepatology* 2022, **76:**155.
95. Park M, Yoo JH, Lee YS, Lee HJ: **Lonicera caerulea Extract Attenuates Non-Alcoholic Fatty Liver Disease in Free Fatty Acid-Induced HepG2 Hepatocytes and in High Fat Diet-Fed Mice.** *Nutrients* 2019, **11**.
96. Lu MC, Lee IT, Hong LZ, Ben-Arie E, Lin YH, Lin WT, Kao PY, Yang MD, Chan YC: **Coffeeberry Activates the CaMKII/CREB/BDNF Pathway, Normalizes Autophagy and Apoptosis Signaling in Nonalcoholic Fatty Liver Rodent Model.** *Nutrients* 2021, **13**.
97. Lan T, Yu Y, Zhang J, Li H, Weng Q, Jiang S, Tian S, Xu T, Hu S, Yang G, et al: **Cordycepin Ameliorates Nonalcoholic Steatohepatitis by Activation of the AMP-Activated Protein Kinase Signaling Pathway.** *Hepatology* 2021, **74:**686.
98. Hu M, Zhang D, Xu H, Zhang Y, Shi H, Huang X, Wang X, Wu Y, Qi Z: **Salidroside Activates the AMP-Activated Protein Kinase Pathway to Suppress Nonalcoholic Steatohepatitis in Mice.** *Hepatology* 2021, **74:**3056.
99. Xu K, Liu S, Zhao X, Zhang X, Fu X, Zhou Y, Xu K, Miao L, Li Z, Li Y, et al: **Treating hyperuricemia related non-alcoholic fatty liver disease in rats with resveratrol.** *Biomed Pharmacother* 2019, **110:**844.
100. Li QP, Dou YX, Huang ZW, Chen HB, Li YC, Chen JN, Liu YH, Huang XQ, Zeng HF, Yang XB, et al: **Therapeutic effect of oxyberberine on obese non-alcoholic fatty liver disease rats.** *Phytomedicine* 2021, **85:**153550.
101. Mai W, Xu Y, Xu J, Zhao D, Ye L, Yu G, Wang Z, Lu Q, Lin J, Yang T, et al: **Berberine Inhibits Nod-Like Receptor Family Pyrin Domain Containing 3 Inflammasome Activation and Pyroptosis in Nonalcoholic Steatohepatitis via the ROS/TXNIP Axis.** *Front Pharmacol* 2020, **11:**185.
102. Wu L, Wang Y, Chi G, Shen B, Tian Y, Li Z, Han L, Zhang Q, Feng H: **Morin reduces inflammatory responses and alleviates lipid accumulation in hepatocytes.** *J Cell Physiol* 2019, **234:**19785.
103. Liu G, Cui Z, Gao X, Liu H, Wang L, Gong J, Wang A, Zhang J, Ma Q, Huang Y, et al: **Corosolic acid ameliorates non-alcoholic steatohepatitis induced by high-fat diet and carbon tetrachloride by regulating TGF-beta1/Smad2, NF-kappaB, and AMPK signaling pathways.** *Phytother Res* 2021, **35:**5214.
104. Ding X, Jian T, Li J, Lv H, Tong B, Li J, Meng X, Ren B, Chen J: **Chicoric Acid Ameliorates Nonalcoholic Fatty Liver Disease via the AMPK/Nrf2/NFkappaB Signaling Pathway and Restores Gut Microbiota in High-Fat-Diet-Fed Mice.** *Oxid Med Cell Longev* 2020, **2020:**9734560.
105. Roh E, Hwang HJ, Kim JW, Hong SH, Kim JA, Lee YB, Choi KM, Baik SH, Yoo HJ: **Ginsenoside Mc1 improves liver steatosis and insulin resistance by attenuating ER stress.** *J Ethnopharmacol* 2020, **259:**112927.
106. Zhang J, Ma X, Fan D: **Ginsenoside CK ameliorates hepatic lipid accumulation via activating the LKB1/AMPK pathway in vitro and in vivo.** *Food & Function* 2022, **13:**1153.
107. Xie K, He X, Chen K, Sakao K, Hou DX: **Ameliorative effects and molecular mechanisms of**

vine tea on western diet-induced NAFLD. *Food Funct* 2020, **11:**5976.

108. Gnoni A, Di Chiara Stanca B, Giannotti L, Gnoni GV, Siculella L, Damiano F: **Quercetin Reduces Lipid Accumulation in a Cell Model of NAFLD by Inhibiting De Novo Fatty Acid Synthesis through the Acetyl-CoA Carboxylase 1/AMPK/PP2A Axis.** *Int J Mol Sci* 2022, **23**.
109. Zhou F, Ding M, Gu Y, Fan G, Liu C, Li Y, Sun R, Wu J, Li J, Xue X, et al: **Aurantio-Obtusin Attenuates Non-Alcoholic Fatty Liver Disease Through AMPK-Mediated Autophagy and Fatty Acid Oxidation Pathways.** *Front Pharmacol* 2021, **12:**826628.
110. Pyun DH, Kim TJ, Park SY, Lee HJ, Abd El-Aty AM, Jeong JH, Jung TW: **Patchouli alcohol ameliorates skeletal muscle insulin resistance and NAFLD via AMPK/SIRT1-mediated suppression of inflammation.** *Mol Cell Endocrinol* 2021, **538:**111464.
111. Mohammed HM: **Zingerone ameliorates non-alcoholic fatty liver disease in rats by activating AMPK.** *J Food Biochem* 2022, **46:**e14149.
112. Wu Z, Geng Y, Buist-Homan M, Moshage H: **Scopoletin and umbelliferone protect hepatocytes against palmitate- and bile acid-induced cell death by reducing endoplasmic reticulum stress and oxidative stress.** *Toxicol Appl Pharmacol* 2022, **436:**115858.
113. Zhong M, Yan Y, Yuan H, A R, Xu G, Cai F, Yang Y, Wang Y, Zhang W: **Astragalus mongholicus polysaccharides ameliorate hepatic lipid accumulation and inflammation as well as modulate gut microbiota in NAFLD rats.** *Food & Function* 2022, **13:**7287.
114. Lee MR, Yang HJ, Park KI, Ma JY: **Lycopus lucidus Turcz. ex Benth. Attenuates free fatty acid-induced steatosis in HepG2 cells and non-alcoholic fatty liver disease in high-fat diet-induced obese mice.** *Phytomedicine* 2019, **55:**14.
115. Zheng Y, Fang D, Huang C, Zhao L, Gan L, Chen Y, Liu F: **Gentiana scabra Restrains Hepatic Pro-Inflammatory Macrophages to Ameliorate Non-Alcoholic Fatty Liver Disease.** *Front Pharmacol* 2021, **12:**816032.
116. Liang M, Huo M, Guo Y, Zhang Y, Xiao X, Xv J, Fang L, Li T, Wang H, Dong S, et al: **Aqueous extract of Artemisia capillaris improves non-alcoholic fatty liver and obesity in mice induced by high-fat diet.** *Front Pharmacol* 2022, **13:**1084435.
117. Zhang X, Song Y, Ding Y, Wang W, Liao L, Zhong J, Sun P, Lei F, Zhang Y, Xie W: **Effects of Mogrosides on High-Fat-Diet-Induced Obesity and Nonalcoholic Fatty Liver Disease in Mice.** *Molecules* 2018, **23**.
118. Wang H, Huang M, Bei W, Yang Y, Song L, Zhang D, Zhan W, Zhang Y, Chen X, Wang W, et al: **FTZ attenuates liver steatosis and fibrosis in the minipigs with type 2 diabetes by regulating the AMPK signaling pathway.** *Biomed Pharmacother* 2021, **138:**111532.
119. Rao Y, Li C, Hu YT, Xu YH, Song BB, Guo SY, Jiang Z, Zhao DD, Chen SB, Tan JH, et al: **A novel HSF1 activator ameliorates non-alcoholic steatohepatitis by stimulating mitochondrial adaptive oxidation.** *Br J Pharmacol* 2022, **179:**1411.
120. Dai L, Wang Q, Wang P, Zhang S, Tai L, Xu X, Sun G, Duan M, Yuan H, Feng Z: **Discovery of novel AdipoRon analogues as potent anti-inflammatory agents against nonalcoholic steatohepatitis.** *Eur J Med Chem* 2022, **244:**114800.
121. Booijink R, Salgado-Polo F, Jamieson C, Perrakis A, Bansal R: **A type IV Autotaxin inhibitor ameliorates acute liver injury and nonalcoholic steatohepatitis.** *EMBO Mol Med* 2022, **14:**e16333.
122. Lin Q, Huang Z, Cai G, Fan X, Yan X, Liu Z, Zhao Z, Li J, Li J, Shi H, et al: **Activating Adenosine Monophosphate-Activated Protein Kinase Mediates Fibroblast Growth Factor 1 Protection**

From Nonalcoholic Fatty Liver Disease in Mice. *Hepatology* 2021, **73:**2206.

123. Zheng Z, Li Y, Fan S, An J, Luo X, Liang M, Zhu F, Huang K: **WW domain-binding protein 2 overexpression prevents diet-induced liver steatosis and insulin resistance through AMPKbeta1.** *Cell Death Dis* 2021, **12:**228.
124. Liu W, Bai F, Wang H, Liang Y, Du X, Liu C, Cai D, Peng J, Zhong G, Liang X, et al: **Tim-4 Inhibits NLRP3 Inflammasome via the LKB1/AMPKalpha Pathway in Macrophages.** *J Immunol* 2019, **203:**990.
125. Argüello RJ, Combes AJ, Char R, Gigan J-P, Baaziz AI, Bousiquot E, Camosseto V, Samad B, Tsui J, Yan P, et al: **SCENITH: A Flow Cytometry-Based Method to Functionally Profile Energy Metabolism with Single-Cell Resolution.** *Cell Metabolism* 2020, **32**.
126. Loomba R, Friedman SL, Shulman GI: **Mechanisms and disease consequences of nonalcoholic fatty liver disease.** *Cell* 2021, **184:**2537.
127. Lomonaco R, Ortiz-Lopez C, Orsak B, Webb A, Hardies J, Darland C, Finch J, Gastaldelli A, Harrison S, Tio F, Cusi K: **Effect of adipose tissue insulin resistance on metabolic parameters and liver histology in obese patients with nonalcoholic fatty liver disease.** *Hepatology (Baltimore, Md)* 2012, **55:**1389.
128. Bhanji RA, Narayanan P, Allen AM, Malhi H, Watt KD: **Sarcopenia in hiding: The risk and consequence of underestimating muscle dysfunction in nonalcoholic steatohepatitis.** *Hepatology (Baltimore, Md)* 2017, **66:**2055.
129. Xu M, Ge C, Zhu L, Qin Y, Du C, Lou D, Li Q, Hu L, Sun Y, Dai X, et al: **iRhom2 Promotes Hepatic Steatosis by Activating MAP3K7-Dependent Pathway.** *Hepatology* 2021, **73:**1346.
130. Wu H, Zhang T, Pan F, Steer CJ, Li Z, Chen X, Song G: **MicroRNA-206 prevents hepatosteatosis and hyperglycemia by facilitating insulin signaling and impairing lipogenesis.** *J Hepatol* 2017, **66:**816.
131. Wang G-X, Zhao X-Y, Lin JD: **The brown fat secretome: metabolic functions beyond thermogenesis.** *Trends In Endocrinology and Metabolism: TEM* 2015, **26:**231.
132. Labruna G, Pasanisi F, Nardelli C, Tarantino G, Vitale DF, Bracale R, Finelli C, Genua MP, Contaldo F, Sacchetti L: **UCP1 -3826 AG+GG genotypes, adiponectin, and leptin/adiponectin ratio in severe obesity.** *Journal of Endocrinological Investigation* 2009, **32:**525.
133. Negi CK, Babica P, Bajard L, Bienertova-Vasku J, Tarantino G: **Insights into the molecular targets and emerging pharmacotherapeutic interventions for nonalcoholic fatty liver disease.** *Metabolism: Clinical and Experimental* 2022, **126:**154925.
134. Malik R, Hodgson H: **The relationship between the thyroid gland and the liver.** *QJM : Monthly Journal of the Association of Physicians* 2002, **95:**559.
135. Harrison SA, Bashir MR, Guy CD, Zhou R, Moylan CA, Frias JP, Alkhouri N, Bansal MB, Baum S, Neuschwander-Tetri BA, et al: **Resmetirom (MGL-3196) for the treatment of non-alcoholic steatohepatitis: a multicentre, randomised, double-blind, placebo-controlled, phase 2 trial.** *Lancet (London, England)* 2019, **394:**2012.
136. Ye P, Xiang M, Liao H, Liu J, Luo H, Wang Y, Huang L, Chen M, Xia J: **Dual-Specificity Phosphatase 9 Protects Against Nonalcoholic Fatty Liver Disease in Mice Through ASK1 Suppression.** *Hepatology* 2019, **69:**76.
137. Lan T, Hu Y, Hu F, Li H, Chen Y, Zhang J, Yu Y, Jiang S, Weng Q, Tian S, et al: **Hepatocyte glutathione S-transferase mu 2 prevents non-alcoholic steatohepatitis by suppressing ASK1 signaling.** *J Hepatol* 2022, **76:**407.

138. Luo X, Li H, Ma L, Zhou J, Guo X, Woo SL, Pei Y, Knight LR, Deveau M, Chen Y, et al: **Expression of STING Is Increased in Liver Tissues From Patients With NAFLD and Promotes Macrophage-Mediated Hepatic Inflammation and Fibrosis in Mice.** *Gastroenterology* 2018, **155:**1971.

139. Li M, Xu C, Shi J, Ding J, Wan X, Chen D, Gao J, Li C, Zhang J, Lin Y, et al: **Fatty acids promote fatty liver disease via the dysregulation of 3-mercaptopyruvate sulfurtransferase/hydrogen sulfide pathway.** *Gut* 2018, **67:**2169.

140. Win S, Min RWM, Zhang J, Kanel G, Wanken B, Chen Y, Li M, Wang Y, Suzuki A, Aung FWM, et al: **Hepatic Mitochondrial SAB Deletion or Knockdown Alleviates Diet-Induced Metabolic Syndrome, Steatohepatitis, and Hepatic Fibrosis.** *Hepatology* 2021, **74:**3127.

141. Anand SK, Caputo M, Xia Y, Andersson E, Cansby E, Kumari S, Henricsson M, Porosk R, Keuenhof KS, Hoog JL, et al: **Inhibition of MAP4K4 signaling initiates metabolic reprogramming to protect hepatocytes from lipotoxic damage.** *J Lipid Res* 2022, **63:**100238.

142. Cui N, Li H, Dun Y, Ripley-Gonzalez JW, You B, Li D, Liu Y, Qiu L, Li C, Liu S: **Exercise inhibits JNK pathway activation and lipotoxicity via macrophage migration inhibitory factor in nonalcoholic fatty liver disease.** *Front Endocrinol (Lausanne)* 2022, **13:**961231.

143. Zhang Z, Wen H, Peng B, Weng J, Zeng F: **CDKN2A deregulation in fatty liver disease and its accelerative role in the process of lipogenesis.** *FASEB J* 2021, **35:**e21230.

144. Jin Y, Tan Y, Zhao P, Guo Y, Chen S, Wu J, Ren Z: **Glutathione S-transferase Mu 2 inhibits hepatic steatosis via ASK1 suppression.** *Commun Biol* 2022, **5:**326.

145. Cho CS, Park HW, Ho A, Semple IA, Kim B, Jang I, Park H, Reilly S, Saltiel AR, Lee JH: **Lipotoxicity induces hepatic protein inclusions through TANK binding kinase 1-mediated p62/sequestosome 1 phosphorylation.** *Hepatology* 2018, **68:**1331.

146. Widjaja AA, Singh BK, Adami E, Viswanathan S, Dong J, D'Agostino GA, Ng B, Lim WW, Tan J, Paleja BS, et al: **Inhibiting Interleukin 11 Signaling Reduces Hepatocyte Death and Liver Fibrosis, Inflammation, and Steatosis in Mouse Models of Nonalcoholic Steatohepatitis.** *Gastroenterology* 2019, **157:**777.

147. Zhang C, Luo X, Chen J, Zhou B, Yang M, Liu R, Liu D, Gu HF, Zhu Z, Zheng H, et al: **Osteoprotegerin Promotes Liver Steatosis by Targeting the ERK-PPAR-gamma-CD36 Pathway.** *Diabetes* 2019, **68:**1902.

148. Yu CJ, Wang QS, Wu MM, Song BL, Liang C, Lou J, Tang LL, Yu XD, Niu N, Yang X, et al: **TRUSS Exacerbates NAFLD Development by Promoting IkappaBalpha Degradation in Mice.** *Hepatology* 2018, **68:**1769.

149. Xu F, Guo M, Huang W, Feng L, Zhu J, Luo K, Gao J, Zheng B, Kong LD, Pang T, et al: **Annexin A5 regulates hepatic macrophage polarization via directly targeting PKM2 and ameliorates NASH.** *Redox Biol* 2020, **36:**101634.

150. Yoo W, Lee J, Noh KH, Lee S, Jung D, Kabir MH, Park D, Lee C, Kwon KS, Kim JS, Kim S: **Progranulin attenuates liver fibrosis by downregulating the inflammatory response.** *Cell Death Dis* 2019, **10:**758.

151. Li Y, Xu J, Lu Y, Bian H, Yang L, Wu H, Zhang X, Zhang B, Xiong M, Chang Y, et al: **DRAK2 aggravates nonalcoholic fatty liver disease progression through SRSF6-associated RNA alternative splicing.** *Cell Metab* 2021, **33:**2004.

152. Song J, Liu Y, Wan J, Zhao GN, Wang JC, Dai Z, Hu S, Yang L, Liu Z, Fu Y, et al: **SIMPLE Is an Endosomal Regulator That Protects Against NAFLD by Targeting the Lysosomal Degradation

of EGFR. *Hepatology* 2021, **74:**3091.
153. Zhang L, Tian R, Yao X, Zhang XJ, Zhang P, Huang Y, She ZG, Li H, Ji YX, Cai J: **Milk Fat Globule-Epidermal Growth Factor-Factor 8 Improves Hepatic Steatosis and Inflammation.** *Hepatology* 2021, **73:**586.
154. Ramani K, Robinson AE, Berlind J, Fan W, Abeynayake A, Binek A, Barbier-Torres L, Noureddin M, Nissen NN, Yildirim Z, et al: **S-adenosylmethionine inhibits la ribonucleoprotein domain family member 1 in murine liver and human liver cancer cells.** *Hepatology* 2022, **75:**280.
155. Li Y, Jin L, Jiang F, Yan J, Lu Y, Yang Q, Zhang Y, Zhang H, Yu H, Zhang Y, et al: **Mutations of NRG4 Contribute to the Pathogenesis of Nonalcoholic Fatty Liver Disease and Related Metabolic Disorders.** *Diabetes* 2021, **70:**2213.
156. Meng D, Pan H, Chen Y, Ding J, Dai Y: **Roles and mechanisms of NRG1 in modulating the pathogenesis of NAFLD through ErbB3 signaling in hepatocytes (NRG1 modulates NAFLD through ErbB3 signaling).** *Obes Res Clin Pract* 2021, **15:**145.
157. Mohammed S, Nicklas EH, Thadathil N, Selvarani R, Royce GH, Kinter M, Richardson A, Deepa SS: **Role of necroptosis in chronic hepatic inflammation and fibrosis in a mouse model of increased oxidative stress.** *Free Radic Biol Med* 2021, **164:**315.
158. Su W, Wu S, Yang Y, Guo Y, Zhang H, Su J, Chen L, Mao Z, Lan R, Cao R, et al: **Phosphorylation of 17beta-hydroxysteroid dehydrogenase 13 at serine 33 attenuates nonalcoholic fatty liver disease in mice.** *Nat Commun* 2022, **13:**6577.
159. Zhao P, Sun X, Chaggan C, Liao Z, In Wong K, He F, Singh S, Loomba R, Karin M, Witztum JL, Saltiel AR: **An AMPK-caspase-6 axis controls liver damage in nonalcoholic steatohepatitis.** *Science (New York, NY)* 2020, **367:**652.
160. Zhang X, Liu S, Zhang C, Zhang S, Yue Y, Zhang Y, Chen L, Yao Z, Niu W: **The role of AMPKalpha2 in the HFD-induced nonalcoholic steatohepatitis.** *Biochim Biophys Acta Mol Basis Dis* 2020, **1866:**165854.
161. Gao H, Zhou L, Zhong Y, Ding Z, Lin S, Hou X, Zhou X, Shao J, Yang F, Zou X, et al: **Kindlin-2 haploinsufficiency protects against fatty liver by targeting Foxo1 in mice.** *Nat Commun* 2022, **13:**1025.

17718536_TiansuLv.docx

ORIGINALITY REPORT

8%
SIMILARITY INDEX

PRIMARY SOURCES

1	www.mdpi.com<br>Internet	36 words — 1%
2	Kim Huard, Aaron C. Smith, Gregg Cappon, Robert L. Dow et al. "Optimizing the Benefit/Risk of Acetyl-CoA Carboxylase Inhibitors through Liver Targeting", Journal of Medicinal Chemistry, 2020<br>Crossref	23 words — < 1%
3	www.wjgnet.com<br>Internet	17 words — < 1%
4	Solji Lee, Daewon Han, Hyun-Goo Kang, Su Jin Jeong, Jae-Eun Jo, Jongdae Shin, Do Kyung Kim, Hwan-Woo Park. "Intravenous sustained-release nifedipine ameliorates nonalcoholic fatty liver disease by restoring autophagic clearance", Biomaterials, 2019<br>Crossref	15 words — < 1%
5	synapse.koreamed.org<br>Internet	13 words — < 1%
6	www.physiology.org<br>Internet	13 words — < 1%
7	pubmed.ncbi.nlm.nih.gov<br>Internet	12 words — < 1%

8 Amir Mohamed Abdelhamid, Ahmed Ramadan Elsheakh, Rania Ramadan Abdelaziz, Ghada Mohamed Suddek. "Empagliflozin ameliorates ethanol-induced liver injury by modulating NF-κB/Nrf-2/PPAR-γ interplay in mice", Life Sciences, 2020 — 11 words — < 1%
Crossref

9 Qing Yao, Xiaohao Wu, Chu Tao, Weiyuan Gong, Mingjue Chen, Minghao Qu, Yiming Zhong, Tailin He, Sheng Chen, Guozhi Xiao. "Osteoarthritis: pathogenic signaling pathways and therapeutic targets", Signal Transduction and Targeted Therapy, 2023 — 11 words — < 1%
Crossref

10 Mengkai Lu, Lei Zhang, Jinyuan Pan, Huishan Shi, Muxin Zhang, Chao Li. "Advances in the study of vascular protective effects and molecular mechanisms of hawthorn (CrataegusanamesaSarg) extracts in cardiovascular diseases", Food & Function, 2023 — 10 words — < 1%
Crossref

11 Yoon-Mi Han, Yong-Jik Lee, Yoo-Na Jang, Hyun-Min Kim, Hong Seog Seo, Tae Woo Jung, Ji Hoon Jeong. " Aspirin Improves Nonalcoholic Fatty Liver Disease and Atherosclerosis through Regulation of the PPAR -AMPK-PGC-1 Pathway in Dyslipidemic Conditions ", BioMed Research International, 2020 — 10 words — < 1%
Crossref

12 qmro.qmul.ac.uk — 10 words — < 1%
Internet

13 warm.dovepress.com — 10 words — < 1%
Internet

14 www.researchgate.net — 10 words — < 1%
Internet

15 Fahimeh Zamani-Garmsiri, Ghasem Ghasempour, Masoume Aliabadi, Seyyed Mohammad Reza Hashemnia et al. "Combination of metformin and chlorogenic acid attenuates hepatic steatosis and inflammation in high-fat diet fed mice", IUBMB Life, 2020 — 9 words — < 1%
Crossref

16 Hui Xiang, Dating Sun, Xin Liu, Zhi-Gang She, Yonghong Chen. "The Role of the Intestinal Microbiota in Nonalcoholic Steatohepatitis", Frontiers in Endocrinology, 2022 — 9 words — < 1%
Crossref

17 Ji-Won Park, Sung-Eun Kim, Na Young Lee, Jung-Hee Kim et al. "Role of Microbiota-Derived Metabolites in Alcoholic and Non-Alcoholic Fatty Liver Diseases", International Journal of Molecular Sciences, 2021 — 9 words — < 1%
Crossref

18 Mechanisms of Insulin Action, 2007. — 9 words — < 1%
Crossref

19 Mohamad Taleuzzaman, Rishabh Verma, Chandra Kala, Parul Sharma, Dipak Kumar Gupta. "Chapter 17 Beneficial Effects of Honey Flavonoids in Nonalcoholic Fatty Liver Disease: An Update", Springer Science and Business Media LLC, 2020 — 9 words — < 1%
Crossref

20 Vidyasagar Naik Bukke, Archana Moola, Gaetano Serviddio, Gianluigi Vendemiale, Francesco Bellanti. "Nuclear factor erythroid 2-related factor 2-mediated signaling and metabolic associated fatty liver disease", World Journal of Gastroenterology, 2022 — 9 words — < 1%
Crossref

21 molmed.biomedcentral.com
Internet
9 words — < 1%

22 static.frontiersin.org
Internet
9 words — < 1%

23 www.e-cmh.org
Internet
9 words — < 1%

24 F. Anthony Romero, Christopher T. Jones, Yingzi Xu, Martijn Fenaux, Randall L. Halcomb. "The Race to Bash NASH: Emerging Targets and Drug Development in a Complex Liver Disease", Journal of Medicinal Chemistry, 2020
Crossref
8 words — < 1%

25 Fabio Nascimbeni, Stefano Ballestri, Mariana Verdelho Machado, Alessandro Mantovani et al. "Clinical relevance of liver histopathology and different histological classifications of NASH in adults", Expert Review of Gastroenterology & Hepatology, 2017
Crossref
8 words — < 1%

26 Guilherme R. Romualdo, Letícia Cardoso Valente, Jéssica Luri Hisano de Souza, Josias Rodrigues, Luís Fernando Barbisan. "Modifying effects of 2,4-D and Glyphosate exposures on gut-liver-adipose tissue axis of diet-induced non-alcoholic fatty liver disease in mice", Ecotoxicology and Environmental Safety, 2023
Crossref
8 words — < 1%

27 T.M., Karthikeya. "A Study of Hepatic Derangements in Type 2 Diabetes", Rajiv Gandhi University of Health Sciences (India), 2023
ProQuest
8 words — < 1%

28 Valvano, Celina M.. "Western Diet-Like Culture Conditions and Oxidative Stress on a Cell Model of
8 words — < 1%

Non-Alcoholic Fatty Liver Disease", The University of Western Ontario (Canada), 2023
ProQuest

29 Wentong Zhang, Jun Wang, Luanfeng Wang, Renjie Shi, Chuanqi Chu, Zhiling Shi, Pujie Liu, Yitong Li, Xuebo Liu, Zhigang Liu. "Alternate-day fasting prevents non-alcoholic fatty liver disease and working memory impairment in diet-induced obese mice", The Journal of Nutritional Biochemistry, 2022 — 8 words — < 1%
Crossref

30 pure.rug.nl — 8 words — < 1%
Internet

31 www.science.gov — 8 words — < 1%
Internet

32 www.xiahepublishing.com — 8 words — < 1%
Internet

33 Alcoholic and Non-Alcoholic Fatty Liver Disease, 2016. — 6 words — < 1%
Crossref

34 Juanjuan Zhao, Mengmeng Guo, Yaping Yan, Ya Wang et al. "The miR-7/EGFR axis controls the epithelial cell immunomodulation and regeneration and orchestrates the pathology in inflammatory bowel disease", Journal of Advanced Research, 2023 — 6 words — < 1%
Crossref

35 Liske M. Kotzé-Hörstmann, Hanél Sadie-Van Gijsen. "Modulation of Glucose Metabolism by Leaf Tea Constituents: A Systematic Review of Recent Clinical and Pre-clinical Findings", Journal of Agricultural and Food Chemistry, 2020 — 6 words — < 1%
Crossref

36	Wendi Teng, Liyun Zhao, Songtao Yang, Chengying Zhang et al. "The hepatic-targeted, resveratrol loaded nanoparticles for relief of high fat diet-induced nonalcoholic fatty liver disease", Journal of Controlled Release, 2019<br>Crossref	6 words — < 1%

EXCLUDE QUOTES	ON	EXCLUDE SOURCES	OFF
EXCLUDE BIBLIOGRAPHY	ON	EXCLUDE MATCHES	OFF
